# Analysis of factors affecting the clinical efficacy and quality of life in the treatment of pediatric acute lymphoblastic leukemia

**DOI:** 10.12669/pjms.40.5.8619

**Published:** 2024

**Authors:** Dan Liu, Yi-fei Zhang

**Affiliations:** 1 Dan Liu, Department of Neonatology, Associate Chief Physician, Associate Professor, The First Affiliated Hospital of Yangtze University, Jingzhou 434000, Hubei, China. The First Clinical Medical Collage of Yangtze University, Jingzhou 434000, Hubei, China. The First Affiliated Hospital of Yangtze University, Jingzhou 434000, Hubei, China; 2 Yi-fei Zhang, Department of Pediatric Medicine, Chief Physician, The First Affiliated Hospital of Yangtze University, Jingzhou 434000, Hubei, China

**Keywords:** Children, Acute lymphoblastic leukemia, Long-term, Clinical efficacy, quality of life

## Abstract

**Objective:**

To analyze the factors affecting the long-term clinical efficacy and quality of life in the treatment of pediatric acute lymphoblastic leukemia (ALL).

**Methods:**

This is a retrospective study. One hundred children with ALL were collected before June, 2018 at The First Affiliated Hospital of Yangtze University and followed up for five years. Not only were their five-years survival rates analyzed, but univariate and multivariate analyses were also performed for factors that might affect their five-year survival rates. The MOS 36-Item Short Form of Health Survey (SF-36) was utilized to investigate the surviving children after five years in order to analyze the factors that may affect the quality of life of the children.

**Results:**

The five-years survival rate of one hundred children with ALL after treatment was 91.00% (91/100). Univariate and multivariate Logistic regression analyses were performed on the factors that may affect the long-term efficacy of pediatric ALL. The results showed that white blood cell count at first diagnosis, prednisone response test, treatment compliance and recurrence were independent risk factors for the long-term efficacy of pediatric ALL(p<0.05). The SF-36 survey of 91 surviving children after five years showed that prednisone response test and treatment compliance were independent risk factors affecting the quality of life of pediatric ALL(p<0.05).

**Conclusion:**

In the initial diagnosis of pediatric ALL, sufficient attention and control should be given to the factors that may affect the long-term clinical efficacy and quality of life, and appropriate treatment plans should be adopted. Meanwhile, the treatment compliance of children should be improved during treatment to improve the survival rate and quality of life of pediatric ALL.

## INTRODUCTION

Acute lymphoblastic leukemia (ALL), the most common hematological malignancy in hematology and pediatrics, mostly makes inroads on children under nine years of age. ALL is clinically manifested as symptoms such as infection, fever, hemorrhage, and anemia.^1^ It poses a serious threat to the health of children as it progresses, and even leads to death in severe cases.^2^-^4^ Currently, continuous improvements are being made in the treatment of ALL. About 95% of the children were in complete remission after treatment, achieving a long-term disease-free survival rate of over 80%.

In high-income countries, the five-years survival rate of children with ALL exceeds 90%.^5^,^6^ Increasingly, ALL is being evaluated not only for the clinical efficacy and survival time of patients but also for the quality of life after treatment. Children are in a critical period of physiological, emotional and role adaptation because of their immature mental development. To make matters worse, the side effects of the ALL diagnosis and treatment will have varying degrees of impact, resulting in lower quality of life for children with ALL than adults. The result has been confirmed by many domestic and foreign studies.^7^-^9^ Children with ALL will not only suffer pain themselves but also bring varying degrees of torture to their families and caregivers. Not only do they have to endure a painful and lengthy process of treatment and rehabilitation, but there is also an uncertain prognosis.

Their family members, especially caregivers, also suffer psychological and emotional blows and face prolonged mental torture. All these factors stand in the way of more favorable treatment outcomes and quality of life for children with ALL. In this study, factors affecting the clinical efficacy and quality of life of children with ALL were analyzed, with a view to providing a clinical reference for improving the clinical efficacy and quality of life of such children.

## METHODS

This is a retrospective study. A total of one hundred hildren collected before June, 2018 with ALL who met the inclusion and exclusion criteria admitted to The First Affiliated Hospital of Yangtze University and were followed up from June 2018 to July 2023. In this study, the clinical data of 100 children with ALL were included, including 59 males and 41 females, aged 2-13 years. All children were diagnosed for the first time by clinical, hematological, and pathological examinations. No relevant treatment was performed before the diagnosis, and malignant tumors originating from other tissues were excluded.

### Ethical Approval

The study was approved by the Institutional Ethics Committee of The First Affiliated Hospital of Yangtze University (No.: LL2023126; Date: July 22, 2023), and written informed consent was obtained from the participants’ guardians.

### Inclusion criteria:

- Children aged 2-13.
- Children who meet the diagnostic criteria of ALL^10^ in the Diagnostic and Therapeutic Criteria for Hematological Diseases (4^th^ Edition) and are diagnosed with ALL.
- Children who are able to listen or answer questions.
- Children with complete and valid data.
- Children’ guardians with informed consent and voluntary participation in this study.

### Exclusion criteria:

- Children with mental disorders or cognitive deficits.
- Children with combined organ failure.
- Children with incomplete data.

### Sample size calculation

In view of the nine influencing factor variables included in this study, the sample size was 45-90 cases according to the requirement that “the sample size for multivariate statistical analysis should be five to ten times the number of independent variables”, plus 20% of the loss of follow-up, and finally, the sample size was 54-108 cases. To ensure an effective sample size, 100 children were included in this study.

### Follow-up

The follow-up was conducted by telephone, outpatient, etc., and lasted for more than five years from the time of the patient’s first hospitalization and discharge. Survival time: refers to the survival time of children calculated on a monthly basis from the starting point of follow-up until death, loss of follow-up or the latest follow-up time as the end point of follow-up. The MOS 36-Item Short Form of Health Survey (SF-36) was used to conduct a follow-up survey on children with a survival period of more than five years at the fifth year. Moreover, the factors that may affect the five-years survival rate and quality of life of the children were analyzed, including gender, age, pathological type, duration of disease, risk, first admission, recurrence, treatment mode and treatment stage.

### Statistical analysis

All data in this study were analyzed using SPSS21.0 software. The measurement data were expressed as mean±standard deviation (*χ̅*±S), and *t* test was used for comparison between groups. Enumeration data were expressed as the number of cases/percentages [n (%)], and comparison between groups was performed by c^2^ test or Fisher’s exact probability method. The sample size is estimated by 95% confidence interval. Logistic regression was performed to analyze the influencing factors, with p<0.05 indicating a statistically significant difference.

## RESULTS

The five-years survival rate of 100 children with ALL after treatment was 91.00% (91/100). Univariate analysis was performed on the factors that may affect the long-term efficacy of pediatric ALL. The results showed that white blood cell count at first diagnosis, cell morphological type, immunoassay, prednisone response test and treatment compliance were all influential factors for the five-years survival rate of the children (p<0.05), Table-I.

**Table-I:** Univariate analysis of the long-term efficacy of children with ALL [n(%)].

Factors	Number of cases	5-year survival rate	c^2^	P
Gender			0.001	0.972
Male	55	50 (90.91)		
Female	45	41 (91.11)		
Age (years)			0.742	0.389
≥6	53	47 (88.68)		
<6	47	44 (93.62)		
WBC count at first diagnosis			6.511	0.011
(50-100)×10^9^/L	68	66 (97.06)		
<50 or >100×10^9^/L	32	25 (78.03)		
Peripheral platelet count at first diagnosis			0.852	0.356
≥20×10^9^/L	52	46 (88.46)		
<20×10^9^/L	48	45 (93.75)		
Peripheral blood hemoglobin count at first diagnosis			1.170	0.279
≥60g/L	61	54 (88.52)		
<60g/L	39	37 (94.87)		
Immunoassay			4.925	0.026
B cell type	25	20 (80.00)		
T cell type	75	71 (94.67)		
Prednisone response test			14.601	0.000
Sensitive	81	78 (96.30)		
Insensitive	19	13 (68.42)		
Treatment compliance			17.767	0.000
Compliance	88	84 (95.45)		
Non-compliance	12	7 (58.33)		
Recurrence			0.235	0.628
Recurrence	63	58 (92.06)		
Non-recurrence	37	33 (89.19)		

Logistic regression analysis showed that white blood cell counts at first diagnosis, prednisone response test, treatment compliance and recurrence were independent risk factors for the long-term efficacy of pediatric ALL (p<0.05), while immunoassay was not an independent risk factor (p<0.05), Table-II. The SF-36 survey of 91 surviving children showed that prednisone response test and treatment compliance were independent risk factors affecting the quality of life of pediatric ALL (p<0.05), Table-III. Logistic regression analysis showed that prednisone response test and treatment compliance were independent risk factors affecting the quality of life of pediatric ALL (p<0.05), Table-IV.

**Table-II:** Multivariate Logistic regression analysis of long-term efficacy of pediatric ALL.

Factors	Regression coefficients	Standard error	Waldc^2^	P	OR value	95%CI
WBC count at first diagnosis	-5.046	2.090	5.831	0.016	0.006	0.000-0.387
Prednisone response test	-6.036	2.589	5.436	0.020	0.002	1.497E-5-0.382
Treatment adherence	-3.916	1.676	5.460	0.019	0.020	0.001-0.532
Recurrence	-4.353	2.213	3.869	0.049	0.013	0.000-0.984

**Table-III:** Univariate analysis of factors affecting the quality of life of pediatric ALL (*χ̅*±*S*, points).

Factors	No. of cases	SF-36 score	t	P
Gender			0.475	0.636
Male	50	79.82±8.35		
Female	41	79.00±8.01		
Age (years)			1.692	0.094
≥6	47	78.06±8.59		
<6	44	80.93±7.50		
WBC count at first diagnosis			1.694	0.094
(50-100)×10^9^/L	66	80.33±7.93		
<50 or >100×10^9^/L	25	77.12±8.46		
Peripheral platelet count at first diagnosis			0.736	0.464
≥20×10^9^/L	46	78.83±8.76		
<20×10^9^/L	45	80.09±7.54		
Peripheral blood hemoglobin count at first diagnosis			0.564	0.574
≥60g/L	54	79.85±7.85		
<60g/L	37	78.86±8.68		
Immunoassay			0.061	0.951
B cell type	20	79.55±7.32		
T cell type	71	79.42±8.43		
Prednisone response test			6.579	0.000
Sensitive	78	81.35±6.91		
Insensitive	13	68.08±5.45		
Treatment compliance			2.433	0.017
Compliance	84	80.04±8.05		
Non-compliance	7	72.43±6.40		
Recurrence			1.073	0.286
Recurrence	58	78.76±8.28		
Non-recurrence	33	80.07±7.93		

**Table-IV:** Multivariate Logistic regression analysis of long-term efficacy of pediatric ALL.

Factors	Regression coefficients	Standard error	Waldc^2^	P	OR value	95%CI
Prednisone response test	4.415	1.100	16.100	0.000	82.667	9.567-714.289
Treatment adherence	2.846	0.910	9.793	0.002	17.222	2.897-102.395

## DISCUSSION

In this study, children with ALL were followed up to explore and analyze the influencing factors of long-term efficacy and quality of life in children with ALL. The results of multivariate analysis showed that WBC count at first diagnosis, prednisone response test, treatment compliance and recurrence were independent risk factors affecting the long-term efficacy of children with ALL(p<0.05). Children with low WBC count at first diagnosis, insensitive prednisone response test, poor treatment compliance and recurrence after treatment had poor long-term efficacy and a low five-years survival rate, which was similar to the results reported.^11^,^12^ Children with poor adherence to treatment had poorer long-term outcomes, which were largely attributed to costs associated with treatment.

In China, most children with ALL have average family economic conditions, but the cost of ALL treatment is relatively high. Some children’s families cannot afford ALL treatment due to financial difficulties, resulting in poor compliance and unsatisfactory long-term clinical efficacy. Recurrence is not only the primary cause of ALL treatment failure^13^ but also the main factor affecting clinical efficacy. Recurrence and poor treatment compliance will interact with each other, leading to an aggravated and malignant condition.

ALL is the disease with the highest incidence rate among pediatric malignant tumors in China, making inroads in 35.6 of every one million Chinese children.^14^ It poses a serious threat to the health of children and even leads to death in severe cases. ALL used to be refractory with a high clinical mortality rate, but now it has witnessed a significant increase in the cure rate with the improvement of medical technology and the availability of more treatment methods. Studies have shown^15^ that about 95% of children with ALL are in complete remission after treatment, and the 5-year survival rate in developed countries is greater than 90%^16^, which has also been increased to 80.0%-90.0% in China.^17^

In view of the high heterogeneity of ALL in children, each child with ALL presents its unique clinical characteristics, especially the different clonal subtypes and biological characteristics of ALL, resulting in different responses to treatment and prognosis of children with ALL.^18^ What’s more, the drugs used to treat ALL may affect the physical and mental state of the children, whereas frequent hospitalizations and pain associated with invasive procedures related to treatment may affect the quality of life of the children.^19^ For this reason, evaluating the long-term clinical efficacy and quality of life of children with ALL after treatment is a critical reference index to measure the success of treatment.

Despite a large number of studies at home and abroad on the factors affecting the prognosis of children with ALL, there are still no independent factors that have a clear impact. A study^20^ pointed out that peripheral WBC count at first diagnosis is a relatively independent factor affecting the prognosis of children with ALL, and children with WBC counts exceeding 200×10^9^/L have an extremely poor prognosis. The prednisone response test is of great significance to the prognosis. Prednisone binds to the receptor to induce apoptosis of malignant cells, thereby exerting a therapeutic effect. It has been shown in studies that early prednisone sensitizers have a high survival rate and a low recurrence rate, while non-sensitizers have a low survival rate, a high recurrence rate and a poor prognosis.^21^

Children with ALL and their families bear a heavier psychological and economic burden than healthy children. Therefore, it is of critical importance to assess the quality of life of children with ALL after long-term treatment. Younger children may be less worried about the disease, while older ones have a strong memory of adverse reactions and negative events during treatment, which can affect their quality of life.^22^,^23^

In this study, factors affecting the long-term quality of life of children with ALL were analyzed, and the results showed that prednisone response test and treatment compliance were independent risk factors affecting the quality of life of children with ALL. As mentioned earlier, those who were sensitive to the prednisone response test had a high survival rate and a low recurrence rate, while those who were not sensitive had a low survival rate, a high recurrence rate and a poor prognosis. As a result, those who were sensitive had high long-term quality of life, and vice versa, low quality of life. Children with high treatment compliance had good a clinical treatment effect and a long disease-free survival period, resulting in high quality of life. In contrast, those with poor treatment compliance had poor treatment outcomes, which can have serious negative effects on themselves and their families, resulting in poor quality of life.

### Limitations of this study

Nevertheless, certain limitations can be seen in this study: a small number of observations were included, and few follow-up factors were set. To address this, more samples will be included and follow-up factors will be increased in subsequent studies to further analyze the factors that affect the long-term clinical efficacy and quality of life of children with ALL.

## CONCLUSIONS

White blood cell counts at first diagnosis, prednisone response test, treatment compliance and recurrence are the primary factors affecting the long-term clinical efficacy of children with ALL, among which prednisone response test and treatment compliance are also important factors affecting the long-term quality of life of such children. At the initial diagnosis, medical staff should identify the factors that may affect the long-term clinical efficacy and quality of life of children with ALL, and give corresponding measures to strengthen education, improve compliance with ALL treatment, and reduce recurrence.

### Authors Contributions:

**YZ:** Carried out the studies, participated in collecting data, drafted the manuscript, are responsible and accountable for the accuracy and integrity of the work.

**DL:** Performed the statistical analysis and participated in its design.

**YZ** and **DL:** Participated in acquisition, analysis, or interpretation of data and drafted the manuscript. All authors read and approved the final manuscript.
